# Temporary flow arrest using EMBOGUARD™ balloon guide catheter may improve functional outcomes in acute stroke thrombectomy: a multicenter retrospective study

**DOI:** 10.3389/fneur.2026.1875382

**Published:** 2026-08-24

**Authors:** Rami Z. Morsi, Sophia Falk, Elena Badillo Goicoechea, Taraf Jaro, Archit B. Baskaran, Sonam Thind, Khalid Trad, Ahmad Khaldi, Reem El-Ghawanmeh, Violiza Inoa, Mohammad H. AlMajali, Amit Chaudhari, Yazan Ashouri, Osama O. Zaidat, Mahmood Mirza, Rachel Mehendale, James E. Siegler, Ali Mansour, Shyam Prabhakaran, Tareq Kass-Hout

**Affiliations:** 1 Department of Neurology, University of Chicago, Chicago, IL, United States; 2 Department of Public Health Sciences, University of Chicago, Chicago, IL, United States; 3 Department of Neurosurgery, Wellstar Health System, Marietta, GA, United States; 4 Department of Neurology, University of Tennessee Health Science Center, Memphis, TN, United States; 5 Neuroscience and Stroke Program, Bon Secours Mercy Health St Vincent Hospital, Toledo, OH, United States; 6 Research Department, Johnson & Johnson Medtech Neurovascular, Galway, Ireland

**Keywords:** balloon guide catheter, flow arrest, ischemic stroke, retrospective, thrombectomy

## Abstract

**Background:**

Balloon guide catheters (BGCs) have demonstrated benefits in mechanical thrombectomy (MT) for acute ischemic stroke (AIS), including improved recanalization rates and reduced distal embolization. The EMBOGUARD™ balloon guide catheter (Johnson & Johnson Neurovascular, Irvine, CA) represents a novel design with enhanced flexibility and compatibility with large-bore aspiration catheters. This study aims to evaluate the safety and efficacy of the EMBOGUARD™ BGC compared with non-BGC approaches.

**Methods:**

We conducted a retrospective multicenter study to analyze patients with AIS due to anterior large vessel occlusions (LVOs) who underwent MT between January 2022 and December 2024. Patients were divided into two groups: those treated with t☺he EMBOGUARD™ BGC and those treated without BGC. Primary outcomes included first-pass effect (FPE), successful recanalization (mTICI ≥2b), procedural time, number of passes, and distal embolization. Secondary outcomes included 90-day functional outcome (modified Rankin Scale [mRS] 0–2), mortality, and symptomatic intracranial hemorrhage (sICH).

**Results:**

A total of 178 patients were included (EMBOGUARD: *n* = 135, 75.8%; non-BGC: *n* = 43, 24.2%). The EMBOGUARD™ group demonstrated comparable rates of FPE (33.3% vs. 37.2%, *p* = 0.68) and successful recanalization (86.7% vs. 86.0%, *p* = 0.92). Procedural time was significantly shorter in the EMBOGUARD™ group (median 32 min vs. 49 min, *p* = 0.004). Distal embolization rates were similar between groups (4.4% vs. 4.7%, *p* = 1.00). The EMBOGUARD™ group showed significantly higher rates of favorable functional outcome at 90 days (48.6% vs. 28.1%, *p* = 0.04) and non-significantly lower 90-day mortality (13.7% vs. 16.7%, *p* = 0.6). Although favorably trending, multivariate logistic regression analysis did not confirm that EMBOGUARD™ was associated with greater odds of FPE (adjusted odds ratio [aOR] 2.01, 95% confidence interval [CI] 0.65–6.41, *p* = 0.2), mRS 0–2 at 90 days (aOR 1.36, 95% CI 0.35–5.34, *p* = 0.7) or lower odds of mortality at 90 days (aOR 0.32, 95% CI 0.05–1.85, *p* = 0.2).

**Conclusion:**

The EMBOGUARD™ balloon guide catheter demonstrated comparable technical efficacy with reduced procedural times and improved clinical outcomes compared with non-BGC techniques in endovascular thrombectomy for acute ischemic stroke. These findings support the use of EMBOGUARD™ as a reasonable tool for flow arrest in stroke intervention.

## Introduction

Mechanical thrombectomy (MT) has become the standard of care for acute ischemic stroke secondary to emergent large vessel occlusions (LVOs), with multiple randomized controlled trials demonstrating superior outcomes compared with medical management alone (1–3). Successful recanalization, particularly achieving first-pass effect (FPE) with minimal passes, correlates strongly with improved functional outcomes (4, 5). Additionally, shorter procedural times have been associated with better neurologic recovery in patients undergoing endovascular therapy (6).

Various technical approaches have evolved to optimize revascularization, including contact aspiration, stent retriever devices, and combined techniques (7–9). Among adjunctive strategies, balloon guide catheters (BGCs) have gained prominence for their potential to enhance recanalization rates, reduce procedural times, and minimize complications (10–12). By providing proximal flow arrest during thrombus retrieval, BGCs theoretically reduce distal embolization and facilitate more complete clot extraction, thereby increasing the likelihood of achieving FPE (13). Despite these theoretical advantages, equipoise exists regarding the routine use of BGCs in MT (14). Concerns include the larger profile of traditional BGCs, which may increase vascular access complications, and challenges in navigating tortuous vascular anatomy (13, 15). Furthermore, temporary flow arrest in patent distal vessels may potentially exacerbate ischemic injury in certain clinical scenarios (16).

The EMBOGUARD™ balloon guide catheter (Johnson & Johnson Neurovascular, Irvine, CA) represents a novel design addressing several limitations of traditional BGCs. With an 8F outer diameter (2.8 mm) and 6.6F (0.087 inch) inner diameter, EMBOGUARD™ provides compatibility with large-bore aspiration catheters while maintaining a relatively low profile. The catheter features enhanced flexibility through its reinforced stainless steel braid construction and hydrophilic coating, potentially facilitating navigation through challenging vascular anatomy. The compliant balloon is manufactured from a polyblend material, expanding concentrically for providing temporary flow arrest. Available in lengths ranging from 85 to 95 cm, EMBOGUARD™ incorporates a non-coaxial balloon inflation lumen to maximize working channel diameter.

Despite the growing body of evidence supporting BGC use in LVOs, data specifically evaluating the EMBOGUARD™ system remain limited. This retrospective multicenter study aimed to compare technical and clinical outcomes between patients treated with the EMBOGUARD™ balloon guide catheter and those treated without BGCs during MT for AIS.

## Methods

### Study design and patient population

This retrospective observational study was conducted across multiple comprehensive stroke centers between January 2022 and December 2024. The study protocol received Institutional Review Board approval at each participating site, with waiver of informed consent granted given the retrospective nature and use of de-identified data.

Patients were included if they met the following criteria: (1) age ≥18 years; (2) acute ischemic stroke with National Institutes of Health Stroke Scale (NIHSS) score ≥5; (3) last known well time to procedure start ≤24 h; (4) baseline modified Rankin Scale (mRS) ≤ 1; and (5) LVO involving the internal carotid artery beyond the petrous segment or middle cerebral artery confirmed on initial imaging. Exclusion criteria comprised: (1) tandem ipsilateral proximal extracranial stenosis/occlusion requiring treatment; (2) previous stent placement in the target lesion; (3) steno-occlusive disease related to intracranial atherosclerotic disease, defined as ≥50% stenosis or occlusion identified on angiogram; (4) Alberta Stroke Program Early CT Score (ASPECTS) ≤ 5 on pre-treatment imaging; (5) intracranial hemorrhage within 30 days; and (6) untreated chronic subdural hematoma >5 mm thickness.

Patients were categorized into two groups based on the use of the EMBOGUARD™ balloon guide catheter during thrombectomy. The decision to use EMBOGUARD™ versus no BGC was at the discretion of the treating neurointerventionalist and based on institutional protocols, patient anatomy, and operator preference. All cases represent consecutive thrombectomy procedures. Group assignment was based on the intended guide catheter strategy at the outset of each procedure, consistent with an intention-to-treat approach.

### Intervention and procedural details

All MT procedures were performed via femoral or radial arterial access under general anesthesia or monitored anesthesia care according to institutional protocols. Thrombectomy techniques included contact aspiration alone, stent retriever alone, or combined approaches, selected at the operator’s discretion. When EMBOGUARD™ was utilized, the balloon was inflated proximally during device retrieval to achieve temporary flow arrest. Intravenous thrombolysis with tissue plasminogen activator was administered according to guideline recommendations when patients met eligibility criteria. All treating neurointerventionalists had a minimum of 10 years of experience in endovascular stroke intervention at the time of the study period.

### Outcome measures

Primary outcomes included: (1) first-pass effect, defined as modified Thrombolysis in Cerebral Infarction (mTICI) score ≥2c after a single pass without adjunctive treatment; (2) successful recanalization, defined as final mTICI score ≥2b; (3) final mTICI score ≥2c; (4) procedural time, measured from groin puncture to final angiogram demonstrating reperfusion; (5) number of device passes; and (6) distal embolization, defined as emboli to previously uninvolved cerebral territories.

Secondary outcomes comprised: (1) functional independence, defined as mRS score 0–2 at 90 days; (2) 90-day mortality from any cause; and (3) symptomatic intracranial hemorrhage (sICH) within 36 h, defined as parenchymal hemorrhage type 2 (PH2) with ≥30% infarct zone involvement and worsening of ≥4 NIHSS points attributable to hemorrhage according to SITS-MOST criteria (17, 18). Angiographic outcomes were assessed by treating interventionalists using the mTICI scale. Clinical outcomes were evaluated through structured telephone interviews or in-person assessments at 90 days by certified stroke coordinators blinded to treatment allocation when feasible.

### Statistical analysis

Baseline characteristics were summarized using descriptive statistics. Continuous variables were presented as median with interquartile range (IQR) and compared using the Wilcoxon rank-sum test. Categorical variables were expressed as frequencies with percentages and compared using chi-squared or Fisher’s exact test as appropriate.

Multivariable regression analyses were performed to adjust for potential confounders including age, sex, race, baseline NIHSS score, ASPECTS value, atrial fibrillation, hypertension, hyperlipidemia, diabetes mellitus, history of ischemic stroke, premorbid mRS, occlusion location, and intravenous thrombolytic use. Odds ratios (OR) with 95% confidence intervals (CI) were calculated, and a two-sided *p*-value <0.05 was considered statistically significant. All statistical analyses were performed using R software version 4.3.1 (R Foundation for Statistical Computing, Vienna, Austria). Sensitivity analyses were also performed, accounting for participating site.

## Results

### Baseline characteristics

**Figure 1:**
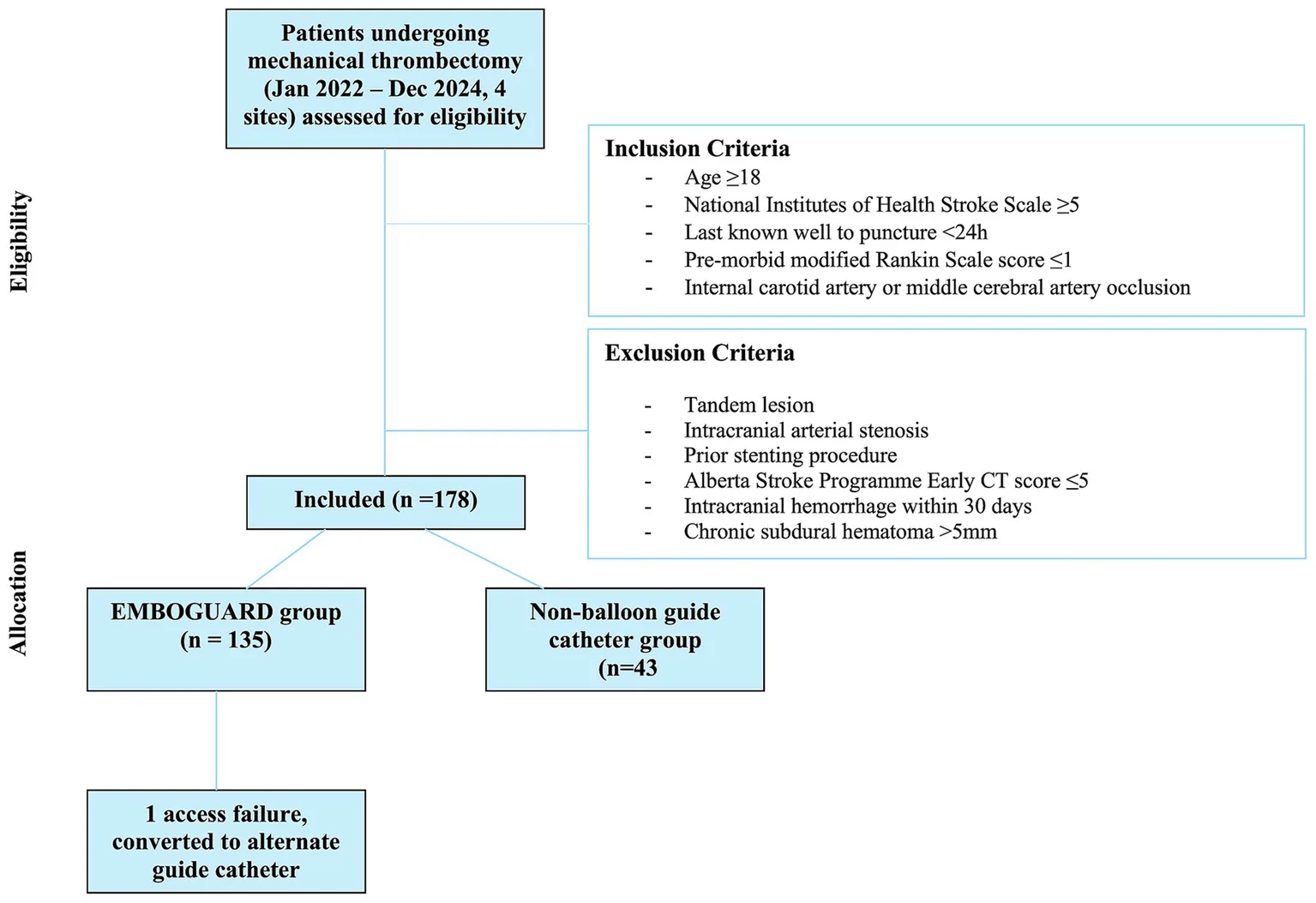
Study flow diagram.

A total of 178 patients underwent mechanical thrombectomy during the study period and met inclusion criteria. Of these, 135 patients (75.8%) were treated with the EMBOGUARD™ balloon guide catheter, and 43 patients (24.2%) were treated without a BGC (Figure 1). Baseline demographic, clinical, and procedural characteristics are summarized in Table 1. The median age was 66 years (IQR 54–76) in the EMBOGUARD™ group compared with 58 years (IQR 48–70) in the non-BGC group. Male sex represented 44.4% of the EMBOGUARD™ group and 53.5% of the non-BGC group. Vascular risk factors were generally balanced between groups, with hypertension present in 66.7% versus 65.1%, hyperlipidemia in 48.1% versus 32.6%, diabetes mellitus in 22.2% versus 20.9%, and atrial fibrillation in 33.3% versus 20.9% of EMBOGUARD™ and non-BGC patients, respectively.

**Table 1:** Baseline patient and procedural characteristics (*n* = 178).

Characteristic	EMBOGUARD (*n* = 135)	Non-BGC (*n* = 43)	*p*-value
Age, years	66.0 (54.5–76.0)	58.0 (48.5–69.5)	0.037
Male sex	60 (44.4)	23 (53.5)	0.390
Race			—
Asian	1 (0.7)	3 (7.0)	
Black/African American	47 (34.8)	32 (74.4)	
White	76 (56.3)	6 (14.0)	
Other	10 (7.4)	1 (2.3)	
Hispanic/Latino ethnicity	104 (77.0)	14 (32.6)	0.000
Comorbidities			
Hypertension	90 (66.7)	28 (65.1)	0.998
Hyperlipidemia	65 (48.1)	14 (32.6)	0.106
Diabetes mellitus	30 (22.2)	9 (20.9)	1.000
Atrial fibrillation	45 (33.3)	9 (20.9)	0.177
Coronary artery disease	23 (17.0)	5 (11.6)	0.543
Congestive heart failure	22 (16.3)	7 (16.3)	1.000
Chronic kidney disease	11 (8.1)	2 (4.7)	0.737
Active malignancy	3 (2.2)	3 (7.0)	0.153
History of ischemic stroke	21 (15.6)	6 (14.0)	0.991
History of TIA	5 (3.7)	1 (2.3)	1.000
History of ICH	0 (0.0)	0 (0.0)	1.000
Premorbid mRS			0.155
0	106 (78.5)	29 (67.4)	
1	27 (20.0)	11 (25.6)	
NR	2 (1.5)	3 (7.0)	
NIHSS upon admission	16 (11–20)	16 (11–20)	0.413
ASPECTS	9 (8–10)	9 (8–10)	0.965
Site of vessel occlusion			—
Internal carotid artery	24 (17.8)	4 (9.3)	
MCA M1	67 (49.6)	20 (46.5)	
MCA M2	44 (32.6)	19 (44.2)	
Time from last known well to puncture, min	325 (208–757)	296 (170–563)	0.398
Initial mTICI score			0.012
0	78 (57.8)	31 (72.1)	
1	0 (0.0)	1 (2.3)	
2a	19 (14.1)	10 (23.3)	
2b	10 (7.4)	1 (2.3)	
2c	20 (14.8)	0 (0.0)	
3	4 (3.0)	0 (0.0)	
General anesthesia	114 (84.4)	31 (72.1)	0.112
Arterial access site			—
Radial	0 (0.0)	16 (37.2)	
Femoral	135 (100.0)	27 (62.8)	
Thrombectomy technique			0.000
Aspiration catheter only	3 (2.2)	10 (23.3)	
Stent retriever only	19 (14.1)	1 (2.3)	
Combination (aspiration + stent retriever)	113 (83.7)	32 (74.4)	
Intravenous thrombolysis	54 (40.0)	16 (37.2)	0.883

Data are presented as *n* (%) for categorical variables and median (interquartile range) for continuous variables. *p*-values were calculated using the Wilcoxon rank-sum test for continuous variables and chi-squared test or Fisher’s exact test for categorical variables, as appropriate. ASPECTS = Alberta Stroke Program Early CT Score; ICA = internal carotid artery; ICH = intracranial hemorrhage; MCA = middle cerebral artery; mRS = modified Rankin Scale; mTICI = modified Thrombolysis in Cerebral Infarction; NIHSS = National Institutes of Health Stroke Scale; TIA = transient ischemic attack.

Baseline stroke severity was comparable, with median NIHSS scores of 16 (IQR 11–20) in both groups. The ASPECTS values were also similar at a median of 9 (IQR 8–10) in both cohorts. The majority of patients had a favorable premorbid functional status. Occlusion location did not significantly differ between groups (*p* = 0.25). Internal carotid artery occlusions occurred in 17.8% of EMBOGUARD patients versus 9.3% of non-BGC patients. Middle cerebral artery (MCA) M1 segment occlusions occurred in 49.6% of EMBOGUARD patients versus 46.5% of non-BGC patients. Occlusions of the MCA M2 segment occurred in 32.6% of EMBOGUARD patients versus 44.2% of non-BGC patients. Intravenous thrombolysis was administered in 40.0% of EMBOGUARD patients and 37.2% of non-BGC patients.

There was one case in which EMBOGUARD placement was attempted but abandoned due to ICA access failure and the procedure was completed with an alternative guide catheter; this patient remained in the EMBOGUARD group for all analyses, in keeping with the intention-to-treat framework. The technical success rate for EMBOGUARD use was >99% (134 of 135 intended cases). Thrombectomy techniques varied slightly between groups. In the EMBOGUARD cohort, combined aspiration and stent retriever approaches were utilized in 83.7% of cases, stent retriever alone in 14.1%, and aspiration alone in 2.2%. In the non-BGC group, combined techniques were used in 74.4% (*n* = 32/43), aspiration alone in 23.3% (*n* = 10/43), and stent retriever alone in 2.3% of procedures (*n* = 1/43). Time from last known well to puncture was similar at 325 min (IQR 208–757) in the EMBOGUARD group and 296 min (IQR 170–563) in the non-BGC group. Baseline mTICI scores differed between groups (*p* = 0.012), driven primarily by occlusion location (Table 1). This imbalance did not confound adjusted analyses, as models controlled for site of occlusion.

### Primary outcomes

Technical and procedural outcomes are presented in Table 2. First-pass effect was achieved in 45 patients (33.3%) in the EMBOGUARD group compared with 16 patients (37.2%) in the non-BGC group (*p* = 0.218). Successful recanalization (mTICI ≥2b) was accomplished in 117 patients (86.7%) with EMBOGUARD and 37 patients (86.1%) without BGC (*p* = 0.917). Complete or near-complete reperfusion (mTICI ≥2c) was achieved in 91 patients (67.4%) and 30 patients (69.8%), respectively (*p* = 0.773).

**Table 2:** Outcomes and procedural complications.

Outcome	EMBOGUARD (*n* = 135)	Non-BGC (*n* = 43)	*p*-value
First-pass effect (mTICI ≥2c after 1 pass)	45 (33.3)	16 (37.2)	0.218
Successful recanalization (mTICI ≥2b)	117 (86.7)	37 (86.1)	0.917
Complete/near-complete reperfusion (mTICI ≥2c)	91 (67.4)	30 (69.8)	0.773
Procedural time, median (IQR), min	32 (23–52)	49 (33–74)	0.004
Number of passes, median (IQR)	2 (1–3)	2 (1–3)	0.555
Procedural complications			
Distal embolization	6 (4.4)	2 (4.7)	1.000
Vessel rupture	0 (0.0)	0 (0.0)	—
Vessel dissection	0 (0.0)	0 (0.0)	—
Iatrogenic carotid-cavernous fistula	0 (0.0)	0 (0.0)	—
Cerebral vasospasm	0 (0.0)	0 (0.0)	—
Access site complication	0 (0.0)	0 (0.0)	—
Favorable functional outcome (mRS 0–2 at 90 days)	53 (48.6)	9 (28.1)	0.040
90-day mortality	16 (13.7)	7 (16.7)	0.636
Symptomatic intracranial hemorrhage	1 (0.7)	0 (0.0)	1.000

Data are presented as *n* (%) for categorical variables and median (interquartile range) for continuous variables. *P*-values were calculated using the Wilcoxon rank-sum test for continuous variables and chi-squared test or Fisher’s exact test for categorical variables, as appropriate. FPE = first-pass effect; IQR = interquartile range; mRS = modified Rankin Scale; mTICI = modified Thrombolysis in Cerebral Infarction; sICH = symptomatic intracranial hemorrhage.

The median number of device passes was 2 (IQR 1–3) in both groups (*p* = 0.555). The full distribution of device passes by group is presented in Table 3. The proportion of cases requiring ≥3 passes was 38.5% in the EMBOGUARD group versus 32.6% in the non-BGC group (*p* = 0.59). The slightly higher crude proportion of ≥3 passes cases in the EMBOGUARD arm is consistent with the higher baseline mTICI ≥2b rate in this group (22.2% vs. 2.3%, *p* = 0.012), which reflects more complex procedural starting conditions and accounts for the unadjusted FPE difference between groups. However, procedural time differed significantly between cohorts: 32 min (IQR 23–52) in the EMBOGUARD group compared with 49 min (IQR 33–74) in the non-BGC group (*p* = 0.004), representing a 35% relative reduction in procedural duration.

**Table 3:** Distribution of device passes per group.

Number of passes	EMBOGUARD (*n* = 135)	Non-BGC (*n* = 43)
1	55 (40.7%)	18 (41.9%)
2	28 (20.7%)	11 (25.6%)
3	19 (14.1%)	8 (18.6%)
4	20 (14.8%)	2 (4.7%)
5+	13 (9.6%)	4 (9.3%)
≥3 passes*	52 (38.5%)	14 (32.6%)

All *p*-values were considered non-significant with values >0.05. *p-value was 0.59.

### Procedural complications

No vessel ruptures, vessel dissections, iatrogenic carotid-cavernous fistulae, cerebral vasospasm events, or access site complications (including groin hematoma and radial artery spasm) occurred in either group. Distal embolization to previously uninvolved territories was observed in 6 patients (4.4%) in the EMBOGUARD group and 2 patients (4.7%) in the non-BGC group (*p* = 1.000). Symptomatic intracranial hemorrhage occurred in 1 patient (0.7%) treated with EMBOGUARD and in no patients in the non-BGC group (*p* = 1.000).

### Secondary outcomes

Among patients with available 90-day follow-up, favorable functional outcome (mRS 0–2 at 90 days) was greater in the EMBOGUARD group with 53 patients (48.6%) compared with 9 patients (28.1%) in the non-BGC group (*p* = 0.040), nearly twice as much. All-cause mortality (at 90 days) occurred in 16 patients (13.7%) in the EMBOGUARD group and 7 patients (16.7%) in the non-BGC group (*p* = 0.636).

### Multivariable analysis

Multivariable logistic regression controlling for site, age, sex, race, baseline NIHSS, ASPECTS, atrial fibrillation, hypertension, hyperlipidemia, diabetes mellitus, history of ischemic stroke, premorbid mRS, occlusion location, and intravenous thrombolytic use is presented in Table 4. EMBOGUARD use was associated with a non-significant two-fold increase in the odds of first-pass effect (adjusted OR 2.01, 95% CI 0.65–6.41, *p* = 0.2), a non-significant reduction in 90-day mortality (adjusted OR 0.32, 95% CI 0.05–1.85, *p* = 0.2), and no significant association with favorable 90-day functional outcome (adjusted OR 1.36, 95% CI 0.35–5.34, *p* = 0.7). Higher baseline NIHSS was independently associated with increased 90-day mortality (adjusted OR 1.18 per point, 95% CI 1.07–1.33, *p* = 0.002) and reduced likelihood of favorable functional outcome (adjusted OR 0.89 per point, 95% CI 0.82–0.96, *p* = 0.004). More distal occlusion location was independently associated with lower odds of first-pass effect (adjusted OR 0.41, 95% CI 0.22–0.73, *p* = 0.003). Sensitivity analyses by occlusion site showed first-pass effect rates for ICA terminus occlusions of 50.0% versus 25.0%, for M1 occlusions of 44.8% versus 45.0%, and for occlusions beyond M1 of 6.8% versus 31.6% in the EMBOGUARD™ and non-BGC groups, respectively. Although these subgroup estimates are imprecise due to small numbers, particularly for ICA terminus occlusions, they suggest that the association between occlusion location and first-pass effect may differ across vascular segments and should be interpreted as hypothesis-generating rather than definitive.

**Table 4:** Multivariable logistic regression analysis results, controlling for site.

Variable	First-pass effect			90-day mortality			Favorable functional outcome at 90 days		
	OR	95% CI	*p*-value	OR	95% CI	*p*-value	OR	95% CI	*p*-value
Use of EMBOGUARD	2.01	0.65, 6.41	0.2	0.32	0.05, 1.85	0.2	1.36	0.35, 5.34	0.7
Age	0.99	0.96, 1.02	0.5	1.01	0.96, 1.07	0.7	1	0.96, 1.04	>0.9
Sex	0.88	0.39, 1.98	0.8	2.21	0.65, 8.34	0.2	0.46	0.20, 1.07	0.075
Race	—	—	—	—	—	—	—	—	—
Asian	—	—	—	—	—	—	—	—	—
Black/African American	0.65	0.04, 8.87	0.7	0.07	0.00, 5.05	0.2	0.18	0.01, 2.49	0.2
White	0.43	0.03, 6.61	0.5	0.13	0.00, 11.0	0.3	0.23	0.01, 3.46	0.3
Other	0.53	0.03, 10.3	0.7	0.09	0.00, 13.1	0.4	1.24	0.06, 24.8	0.9
NIHSS upon admission	0.99	0.92, 1.06	0.8	1.18	1.07, 1.33	0.002	0.89	0.82, 0.96	0.004
ASPECTS value	—	—	—	—	—	—	—	—	—
10	—	—	—	—	—	—	—	—	—
6	0.27	0.02, 1.90	0.2	1.74	0.14, 17.8	0.6	0	—	>0.9
7	2	0.39, 10.7	0.4	1.39	0.14, 11.0	0.8	0.78	0.14, 3.99	0.8
8	0.74	0.25, 2.13	0.6	0.83	0.17, 3.68	0.8	0.45	0.14, 1.36	0.2
9	0.29	0.07, 1.01	0.06	1.22	0.16, 7.83	0.8	0.72	0.18, 2.83	0.6
Atrial fibrillation	0.72	0.27, 1.86	0.5	2.38	0.62, 9.69	0.2	1.01	0.35, 2.89	>0.9
Hypertension	1.2	0.44, 3.31	0.7	1.62	0.32, 8.80	0.6	0.48	0.17, 1.34	0.2
Hyperlipidemia	1.78	0.62, 5.29	0.3	1.65	0.30, 9.51	0.6	1.57	0.51, 5.18	0.4
Diabetes mellitus	3.98	1.46, 11.6	0.008	0.95	0.21, 4.23	>0.9	1.43	0.44, 4.56	0.5
History of ischemic stroke	0.59	0.15, 2.15	0.4	1.36	0.22, 7.82	0.7	1.72	0.47, 6.10	0.4
Premorbid mRS	—	—	—	—	—	—	—	—	—
0	—	—	—	—	—	—	—	—	—
1	1.74	0.57, 5.46	0.3	2.5	0.46, 14.0	0.3	0.76	0.22, 2.51	0.7
Site of intracranial vessel occlusion	0.41	0.22, 0.73	0.003	1.06	0.43, 2.75	>0.9	0.71	0.37, 1.30	0.3
Intravenous thrombolytic use	2.05	0.88, 4.92	0.1	0.71	0.16, 2.87	0.6	2.28	0.96, 5.59	0.064

Multivariable logistic regression models adjusted for listed covariates. ASPECTS = Alberta Stroke Program Early CT Score; CI = confidence interval; FPE = first-pass effect (mTICI ≥2c after single pass); mRS = modified Rankin Scale; NIHSS = National Institutes of Health Stroke Scale; OR = odds ratio. Reference categories: race (Asian), ASPECTS value (10), premorbid mRS (0).

In a sensitivity analysis restricted to sites where both EMBOGUARD and non-BGC approaches were available (Supplementary Table 1), the point estimates for EMBOGUARD use were larger: adjusted OR 3.49 for first-pass effect (95% CI 0.88–15.8, *p* = 0.086), adjusted OR 0.15 for 90-day mortality (95% CI 0.01–1.73, *p* = 0.2), and adjusted OR 5.40 for favorable functional outcome (95% CI 0.78–56.2, *p* = 0.11). However, confidence intervals remained wide, reflecting the reduced sample size.

## Discussion

This multicenter retrospective study of 178 patients undergoing mechanical thrombectomy for acute ischemic stroke due to LVO found that the EMBOGUARD balloon guide catheter achieved comparable recanalization rates with significantly shorter procedural times compared with non-BGC approaches. On unadjusted analysis, the EMBOGUARD group demonstrated a significantly higher rate of favorable 90-day functional outcome (48.6% vs. 28.1%, *p* = 0.040), though this association did not persist after multivariable adjustment. No procedural safety signals were identified. These findings position EMBOGUARD as a technically feasible and safe tool for flow arrest during stroke thrombectomy, while highlighting the need for larger prospective studies to define its clinical benefit.

### Procedural efficiency

The most robust finding of this study was the 35% relative reduction in median procedural time with EMBOGUARD (32 vs. 49 min, *p* = 0.004), which persisted despite equivalent numbers of device passes and similar first-pass effect rates between groups. This is a clinically meaningful observation. Alawieh et al. demonstrated in a multicenter analysis of 1,359 patients that the likelihood of good functional outcome declines when procedural time exceeds 30 min, with rates of symptomatic hemorrhage and complications increasing exponentially beyond that threshold (6). Similarly, Baek et al. reported that even among patients achieving first-pass recanalization, favorable outcomes are not guaranteed when puncture-to-recanalization time exceeds 30 min (19). The procedural time advantage observed with EMBOGUARD may therefore translate into reduced ischemic burden through earlier reperfusion.

This efficiency gain likely reflects the catheter’s design characteristics. The non-coaxial balloon inflation lumen preserves maximal inner diameter (0.087 inches), allowing passage of contemporary large-bore aspiration catheters without the friction or kinking that can slow device exchanges through smaller-lumen BGCs. The hydrophilic distal coating and reinforced stainless steel braid may also facilitate navigation and stable positioning in the distal cervical internal carotid artery, potentially reducing setup time relative to traditional non-BGCs that lack these features.

### Recanalization and first-pass effect

The comparable first-pass effect (33.3% vs. 37.2%) and successful recanalization rates (86.7% vs. 86.1%) between groups indicate that EMBOGUARD did not compromise technical efficacy. The recanalization rates observed in both arms are consistent with benchmarks from contemporary large-vessel occlusion registries and trials (1–3). In contrast to some prior observational data reporting higher FPE with BGC use, such as the STRATIS registry, where BGC-treated patients achieved 48% FPE versus 26–35% with other guide catheter strategies (12). We observed no significant FPE advantage. This is consistent with recent meta-analytic data suggesting that the incremental FPE benefit of BGCs may be modest when combined aspiration-plus-stent-retriever techniques are employed, as was the dominant approach in both arms of our study (20). It is also possible that with advances in aspiration catheter technology and retrieval techniques over the past decade, baseline FPE rates have improved to a degree that limits the marginal gain attributable to proximal flow arrest alone (21). The full pass-distribution analysis confirms that the two groups had comparable single-pass rates (40.7% vs. 41.9%) and that observed similarity in median total passes masks a higher rate of ≥3-pass procedures in the EMBOGUARD, driven by the higher burden of partial baseline reperfusion in that arm. Additionally, the higher baseline mTICI 2b/2c rates in the EMBOGUARD cohort (22.2% vs. 2.3%) reflect preferential BGC use in cases with partial reperfusion, consistent with operator selection for complex anatomy. Adjusted analyses accounting for occlusion location confirmed no independent effect on first-pass effect.

### Safety profile

No vessel ruptures, dissections, iatrogenic carotid-cavernous fistulae, vasospasm events, or access site complications occurred in either group. This is noteworthy given that one concern historically raised about BGCs is the potential for vascular injury from larger-profile devices (15). The absence of access site complications despite 100% femoral access in the EMBOGUARD group (compared with 62.8% femoral and 37.2% radial in the non-BGC group) supports the safety of EMBOGUARD’s 8F profile. Similarly, the low and equivalent distal embolization rates (4.4% vs. 4.7%) are consistent with the expected benefit of proximal flow arrest, though the equivalent rates in the non-BGC group suggest that modern aspiration catheters and retrieval techniques have substantially reduced baseline embolization risk regardless of BGC use (22). The numerically higher count in the EMBOGUARD arm (6 vs. 2 events, *p* = 1.000) is not statistically meaningful at these event counts and is more plausibly explained by the higher proportion of ICA terminus occlusions, greater stent retriever use, and higher ≥3-pass rate in that group, all established independent predictors of distal embolization regardless of catheter type (13, 23, 24).

### Clinical outcomes

On unadjusted analysis, the EMBOGUARD group achieved a significantly higher rate of functional independence at 90 days (48.6% vs. 28.1%, *p* = 0.040). However, after multivariable adjustment controlling for site, baseline stroke severity, age, and other confounders, this difference was no longer statistically significant (adjusted OR 1.36, 95% CI 0.35–5.34, *p* = 0.7). The attenuation of effect upon adjustment likely reflects confounding by measured variables, particularly the significant between-group differences in race, ethnicity, arterial access site, and thrombectomy technique, as well as the limited statistical power inherent to a sample of 178 patients with an unbalanced allocation (135 vs. 43). On the other hand, the nearly two-fold higher unadjusted rate of functional independence despite similar recanalization and procedural safety characteristics may suggest another phenomenon. Several *in vitro* studies have found that BGCs decrease distal embolization with tiny particles smaller than 200 microns and even smaller micro-fragments (25). This suggests a reduction of microvascular sludge due to tiny fragmentation of the clot, undetectable by typical digital subtraction angiography, which could have improved local capillary flow resulted in enhanced tissue salvage. The median ASPECTS was identical between groups (9 in both arms, *p* = 0.965), and ASPECTS was included as an explicit covariate in the adjusted model; the attenuation of the functional outcome signal after adjustment therefore reflects the combined confounding by racial/ethnic composition, access site, and thrombectomy technique rather than baseline infarct extent alone.

The sensitivity analysis restricted to sites that employed both EMBOGUARD and non-BGC approaches yielded a substantially larger point estimate for favorable functional outcome (adjusted OR 5.40, 95% CI 0.78–56.2, *p* = 0.11) and a more pronounced mortality reduction (adjusted OR 0.15, 95% CI 0.01–1.73, *p* = 0.2), suggesting that inter-site practice variation may have obscured a real effect in the primary analysis. However, the extremely wide confidence intervals preclude definitive interpretation and emphasize the need for adequately powered prospective investigation.

### Contextualization within the evolving balloon guide catheter literature

Our findings contribute to a growing but increasingly complex body of evidence on BGC use in stroke thrombectomy. Multiple large registries, including STRATIS (12), NASA (10), TRACK (26), and ROSSETTI (21), and a comprehensive meta-analysis of 5,507 patients by Podlasek et al. (20) have reported that BGC use is associated with improved FPE, reduced procedural times, lower distal embolization, and better functional outcomes. A 17-center Korea registry of 955 patients further demonstrates that BGC benefit extends to both stent retriever and contact aspiration modalities (27). These observational data formed the basis of the European Stroke Organisation–ESMINT guideline recommendation that mechanical thrombectomy be preferably conducted with a proximal BGC (28).

However, the recently published PROTECT-MT trial, the first randomized controlled trial comparing BGC with conventional guide catheters, reported a strikingly different conclusion (29). In this multicenter Chinese trial of 329 patients (terminated early for safety concerns), the BGC group had significantly worse 90-day mRS scores than the conventional guide catheter group (adjusted common OR 0.66, 95% CI 0.45–0.98, *p* = 0.037), with numerically higher 90-day mortality (24% vs. 16%) and significantly more internal carotid artery vasospasm (4% vs. 1%). These findings demand careful consideration. Several design features of PROTECT-MT may limit its generalizability: the BGC type was not standardized across sites, balloon inflation protocols were not uniform, operator experience with BGCs was not reported, and the trial enrolled patients in China where practice patterns and device availability differ from those in North America and Europe. Furthermore, the ProFATE randomized trial (*n* = 134, 4 UK centers) compared balloon inflation versus no inflation with both arms using a BGC *in situ*, thereby isolating the effect of proximal flow arrest itself (30). The primary outcome of near-complete/complete recanalization (eTICI 2c–3) did not differ significantly between groups, though first-pass complete reperfusion was significantly higher in the flow arrest arm and 90-day functional outcomes showed a non-significant trend favoring flow arrest. Taken together, PROTECT-MT and ProFATE highlight the persistent uncertainty surrounding the clinical benefit of proximal flow arrest and underscore the need for larger, well-designed trials, particularly those evaluating newer-generation BGC devices such as EMBOGUARD.

Our data offer a complementary perspective. Unlike PROTECT-MT in which BGC use was associated with longer procedural times (29), EMBOGUARD was associated with shorter procedural times, a discrepancy that may reflect the catheter’s specific design advantages (large inner diameter, hydrophilic coating, and non-coaxial inflation lumen) over older BGC models. Importantly, our study showed no vasospasm events and no vascular access complications in the EMBOGUARD group, contrasting with the excess vasospasm reported in PROTECT-MT. The ProFATE trial found improved but non-significant clinical outcomes in the BGC group, but suffered from a limited sample size (*n* = 134) (30). Collectively, PROTECT-MT and ProFATE suggest that the clinical impact of BGC use is nuanced, potentially device-specific and technique-dependent, and that newer-generation catheter designs prioritizing navigability and inner lumen diameter may mitigate the risks observed with earlier devices while preserving the hemodynamic benefits of flow arrest.

## Limitations

Several limitations should be acknowledged. First, the retrospective observational design is susceptible to selection bias, as the decision to use EMBOGUARD was not randomized. Operators may have preferentially used BGCs in cases with favorable anatomy or more accessible occlusions, potentially inflating apparent benefits. Second, the unbalanced group sizes (135 vs. 43) limit statistical power, particularly for detecting differences in uncommon outcomes such as symptomatic hemorrhage. The wide confidence intervals observed in both the primary and sensitivity multivariable analyses are a direct consequence of this limitation. The unbalanced group allocation was driven by the site selection structure, with the majority of non-BGC cases originating from centers where conventional guide catheters remained institutional standard; this structural imbalance limits statistical power and cannot be fully mitigated by multivariable adjustment. The wide confidence intervals observed in both the primary and sensitivity multivariable analyses are a direct consequence of this limitation. Third, significant between-group differences in race, ethnicity, arterial access site, and thrombectomy technique represent potential confounders that may not be fully mitigated by statistical adjustment. Fourth, angiographic outcomes were adjudicated by treating interventionalists rather than a blinded core laboratory, introducing potential assessment bias. Fifth, 90-day clinical follow-up was not available for all patients, and the resulting smaller denominators for secondary outcomes (109 in EMBOGUARD, 32 in non-BGC with mRS data) may introduce attrition bias. This study cannot determine whether observed differences are attributable to the EMBOGUARD catheter specifically versus the general principle of proximal flow arrest, as the non-BGC group did not employ any BGC rather than a comparator BGC device. Sixth, vascular tortuosity was not formally measured, and the significant between-group difference in radial versus femoral access (37.2% vs. 0%) may partly reflect unmeasured anatomical selection factors that could independently influence procedural time and guide catheter choice. Seventh, territory-specific categorization of distal embolization events was not available in this dataset; future studies should systematically document anterior cerebral artery and MCA territory embolization given its independent prognostic significance. Finally, the exclusion of patients with tandem lesions, intracranial atherosclerotic disease, and ASPECTS ≤5 means findings are most directly applicable to patients with embolic anterior circulation LVOs, and conclusions should be interpreted in that context.

## Future directions

These findings provide rationale for a prospective, randomized evaluation of the EMBOGUARD balloon guide catheter in acute ischemic stroke thrombectomy. Neither PROTECT-MT, which used heterogeneous BGC types across Chinese centers, nor ProFATE, which isolated the effect of balloon inflation in a small UK cohort, evaluated EMBOGUARD or other newer-generation BGC designs specifically. A future trial should incorporate standardized procedural protocols including mandatory balloon inflation during retrieval, blinded core-laboratory adjudication of angiographic outcomes, complete 90-day follow-up, and adequate sample size to detect clinically meaningful differences in functional outcomes. Comparative studies against other contemporary BGC designs (e.g., FlowGate, Walrus, BOBBY, Branchor XF) would further clarify whether the specific engineering features of EMBOGUARD, particularly its large inner diameter and purportedly enhanced navigability, confer measurable advantages beyond those attributable to proximal flow arrest alone.

## Conclusion

In this multicenter retrospective study, the EMBOGUARD balloon guide catheter achieved recanalization rates comparable to non-BGC approaches with significantly shorter procedural times and a favorable safety profile. Although unadjusted analysis suggested higher rates of functional independence at 90 days with EMBOGUARD, this association did not persist after multivariable adjustment, likely reflecting the study’s limited sample size and residual confounding variables. These findings support the technical feasibility and safety of EMBOGUARD for proximal flow arrest during mechanical thrombectomy and warrant prospective randomized evaluation to determine its effect on clinical outcomes.

## Data Availability

The raw data supporting the conclusions of this article will be made available by the authors, without undue reservation.

## References

[1] Goyal M Demchuk AM Menon BK Eesa M Rempel JL Thornton J . Randomized assessment of rapid endovascular treatment of ischemic stroke. N Engl J Med. (2015) 372:1019–30. doi: 10.1056/NEJMoa1414905, 25671798

[2] Jovin TG Nogueira RG Lansberg MG Demchuk AM Martins SO Mocco J . Thrombectomy for anterior circulation stroke beyond 6 h from time last known well (AURORA): a systematic review and individual patient data meta-analysis. Lancet. (2022) 399:249–58. doi: 10.1016/S0140-6736(21)01341-6, 34774198

[3] Saver JL Goyal M Bonafe A Diener HC Levy EI Pereira VM . Stent-retriever thrombectomy after intravenous t-PA vs. t-PA alone in stroke. N Engl J Med. (2015) 372:2285–95. doi: 10.1056/NEJMoa1415061, 25882376

[4] Flottmann F Leischner H Broocks G Nawabi J Bernhardt M Faizy TD . Recanalization rate per retrieval attempt in mechanical thrombectomy for acute ischemic stroke. Stroke. (2018) 49:2523–5. doi: 10.1161/STROKEAHA.118.022737, 30355115

[5] Yoon W Kim SK Park MS Baek BH Lee YY. Predictive factors for good outcome and mortality after stent-retriever thrombectomy in patients with acute anterior circulation stroke. J Stroke. (2017) 19:97–103. doi: 10.5853/jos.2016.00675, 28178407 PMC5307937

[6] Alawieh A Vargas J Fargen KM Langley EF Starke RM De Leacy R . Impact of procedure time on outcomes of thrombectomy for stroke. J Am Coll Cardiol. (2019) 73:879–90. doi: 10.1016/j.jacc.2018.11.052, 30819354

[7] Phan K Maingard J Kok HK Dmytriw AA Goyal S Chandra R . Contact aspiration versus stent-retriever thrombectomy for distal middle cerebral artery occlusions in acute ischemic stroke: Meta-analysis. Neurointervention. (2018) 13:100–9. doi: 10.5469/neuroint.2018.00997, 30196680 PMC6132031

[8] Primiani CT Vicente AC Brannick MT Turk AS Mocco J Levy EI . Direct aspiration versus stent retriever thrombectomy for acute stroke: a systematic review and Meta-analysis in 9127 patients. J Stroke Cerebrovasc Dis. (2019) 28:1329–37. doi: 10.1016/j.jstrokecerebrovasdis.2019.01.034, 30772159

[9] Ye G Wen X Wang H Sun C Pan Z Chen M . First-line contact aspiration versus first-line stent retriever for acute posterior circulation strokes: an updated meta-analysis. J Neurointerv Surg. (2022) 14:457–63. doi: 10.1136/neurintsurg-2021-017497, 34035153

[10] Nguyen TN Malisch T Castonguay AC Gupta R Sun CH Martin CO . Balloon guide catheter improves revascularization and clinical outcomes with the solitaire device: analysis of the north American solitaire acute stroke registry. Stroke. (2014) 45:141–5. doi: 10.1161/STROKEAHA.113.002407 24302483

[11] Velasco A Buerke B Stracke CP Berkemeyer S Mosimann PJ Schwindt W . Comparison of a balloon guide catheter and a non-balloon guide catheter for mechanical thrombectomy. Radiology. (2016) 280:169–76. doi: 10.1148/radiol.2015150575, 26789499

[12] Zaidat OO Mueller-Kronast NH Hassan AE Haussen DC Jadhav AP Froehler MT . Impact of balloon guide catheter use on clinical and angiographic outcomes in the STRATIS stroke thrombectomy registry. Stroke. (2019) 50:697–704. doi: 10.1161/STROKEAHA.118.021126, 30776994

[13] Brinjikji W Starke RM Murad MH Fiorella D Pereira VM Goyal M . Impact of balloon guide catheter on technical and clinical outcomes: a systematic review and meta-analysis. J Neurointerv Surg. (2018) 10:335–9. doi: 10.1136/neurintsurg-2017-013179, 28754806

[14] Powers WJ Rabinstein AA Ackerson T Adeoye OM Bambakidis NC Becker K . Guidelines for the early Management of Patients with Acute Ischemic Stroke: 2019 update to the 2018 guidelines for the early Management of Acute Ischemic Stroke: a guideline for healthcare professionals from the American Heart Association/American Stroke Association. Stroke. (2019) 50:e344–418. doi: 10.1161/STR.0000000000000211, 31662037

[15] Shah VA Martin CO Hawkins AM Holloway WE Junna S Akhtar N. Groin complications in endovascular mechanical thrombectomy for acute ischemic stroke: a 10-year single center experience. J Neurointerv Surg. (2016) 8:568–70. doi: 10.1136/neurintsurg-2015-011763, 26002302 PMC4893108

[16] Orscelik A Kallmes DF Bilgin C Musmar B Senol YC Kobeissi H . Comparison of balloon guide catheter versus non-balloon guide catheter for mechanical thrombectomy in patients with distal medium vessel occlusion. J Neurointerv Surg. (2024) 16:587–94. doi: 10.1136/jnis-2023-020925, 37918906

[17] Mazya M Egido JA Ford GA Lees KR Mikulik R Toni D . Predicting the risk of symptomatic intracerebral hemorrhage in ischemic stroke treated with intravenous alteplase: safe implementation of treatments in stroke (SITS) symptomatic intracerebral hemorrhage risk score. Stroke. (2012) 43:1524–31. doi: 10.1161/STROKEAHA.111.644815 22442178

[18] von Kummer R Broderick JP Campbell BC Demchuk A Goyal M Hill MD . The Heidelberg bleeding classification: classification of bleeding events after ischemic stroke and reperfusion therapy. Stroke. (2015) 46:2981–6. doi: 10.1161/STROKEAHA.115.010049 26330447

[19] Baek JH Heo JH Nam HS Kim BM Kim DJ Kim YD. Clinical benefit of first-pass recanalization is time-dependent in endovascular treatment of acute ischemic stroke. J Clin Med. (2023) 12:6596. doi: 10.3390/jcm12206596, 37892733 PMC10607503

[20] Podlasek A Dhillon PS Jewett G Shahein A Goyal M Almekhlafi M. Clinical and procedural outcomes with or without balloon guide catheters during endovascular thrombectomy in acute ischemic stroke: a systematic review and Meta-analysis with first-line technique subgroup analysis. AJNR Am J Neuroradiol. (2021) 42:1464–71. doi: 10.3174/ajnr.A7164, 34045301 PMC8367631

[21] Blasco J Puig J Daunis IEP Gonzalez E Fondevila Monso JJ Manso X . Balloon guide catheter improvements in thrombectomy outcomes persist despite advances in intracranial aspiration technology. J Neurointerv Surg. (2021) 13:773–8. doi: 10.1136/neurintsurg-2020-017027 33632881

[22] Chueh JY Kuhn AL Puri AS Wilson SD Wakhloo AK Gounis MJ. Reduction in distal emboli with proximal flow control during mechanical thrombectomy: a quantitative in vitro study. Stroke. (2013) 44:1396–401. doi: 10.1161/STROKEAHA.111.670463, 23493730

[23] Ahn JH Cho SS Kim SE Kim HC Jeon JP. The effects of balloon-guide catheters on outcomes after mechanical thrombectomy in acute ischemic strokes: a Meta-analysis. J Korean Neurosurg Soc. (2019) 62:389–97. doi: 10.3340/jkns.2018.0165, 31064042 PMC6616979

[24] Tan Y Mao Z Li Z Fan H. Predictors of distal embolization during thrombectomy for anterior circulation large vessel bifurcation occlusion stroke. J Neurointerv Surg. (2025) 18:121–5. doi: 10.1136/jnis-2024-022415, 39481885 PMC12772570

[25] Chueh JY Puri AS Wakhloo AK Gounis MJ. Risk of distal embolization with stent retriever thrombectomy and ADAPT. J Neurointerv Surg. (2016) 8:197–202. doi: 10.1136/neurintsurg-2014-011491, 25540180 PMC4752657

[26] Nguyen TN Castonguay AC Nogueira RG Haussen DC English JD Satti SR . Effect of balloon guide catheter on clinical outcomes and reperfusion in Trevo thrombectomy. J Neurointerv Surg. (2019) 11:861–5. doi: 10.1136/neurintsurg-2018-014452, 30712011

[27] Baek JH Kim BM Kang DH Heo JH Nam HS Kim YD . Balloon guide catheter is beneficial in endovascular treatment regardless of mechanical recanalization modality. Stroke. (2019) 50:1490–6. doi: 10.1161/STROKEAHA.118.024723, 31043149

[28] Turc G Bhogal P Fischer U Khatri P Lobotesis K Mazighi M . European stroke organisation (ESO) - European Society for Minimally Invasive Neurological Therapy (ESMINT) guidelines on mechanical thrombectomy in acute ischemic stroke. J Neurointerv Surg. (2023) 15:e8. doi: 10.1136/neurintsurg-2018-014569, 30808653

[29] Liu J Zhou Y Zhang L Li Z Chen W Zhu Y . Balloon guide catheters for endovascular thrombectomy in patients with acute ischaemic stroke due to large-vessel occlusion in China (PROTECT-MT): a multicentre, open-label, blinded-endpoint, randomised controlled trial. Lancet. (2024) 404:2165–74. doi: 10.1016/S0140-6736(24)02315-8, 39579782

[30] Dhillon PS Butt W Podlasek A Bhogal P Lynch J Booth TC . Effect of proximal blood flow arrest during endovascular thrombectomy (ProFATE): a multicenter, blinded-end point, randomized clinical trial. Stroke. (2025) 56:371–9. doi: 10.1161/STROKEAHA.124.049715 39697177 PMC11771355

