# Supplementary material for: Temporary flow arrest using EMBOGUARD™ balloon guide catheter may improve functional outcomes in acute stroke thrombectomy: a multicenter retrospective study
Source: Front Neurol. 2026 Aug 24;17:1875382. doi: 10.3389/fneur.2026.1875382 (PMC13547071; doi:10.3389/fneur.2026.1875382)
Supplement: Supplementary file 1 [file Table_1.docx]

| **Supplementary Table 1.** Multivariable logistic regression analysis results with cases only from sites where both EMBOGUARD and non-balloon guide catheter approaches were available. | | | | | | | | | |
| --- | --- | --- | --- | --- | --- | --- | --- | --- | --- |
|  | **First-pass effect** | | | **90-day mortality** | | | **Favorable functional outcome at 90 days** | | |
| **Variable** | **OR** | **95% CI** | **p-value** | **OR** | **95% CI** | **p-value** | **OR** | **95% CI** | **p-value** |
| *Use of EMBOGUARD* | 3.49 | 0.88, 15.8 | 0.086 | 0.15 | 0.01, 1.73 | 0.2 | 5.4 | 0.78, 56.2 | 0.11 |
| *Age* | 1.01 | 0.96, 1.06 | 0.8 | 1.03 | 0.96, 1.14 | 0.4 | 1.03 | 0.96, 1.11 | 0.4 |
| *Sex* | 0.97 | 0.27, 3.46 | >0.9 | 3.04 | 0.17, 127 | 0.5 | 2.26 | 0.39, 15.5 | 0.4 |
| *Race* | — | — | — | — | — | — | — | — | — |
| Asian | — | — | — | — | — | — | — | — | — |
| Black/African American | 0.08 | 0.00, 7.30 | 0.3 | 0.04 | 0.00, 18.4 | 0.4 | 0.08 | 0.00, 13.4 | 0.3 |
| White | 0.01 | 0.00, 1.31 | 0.072 | 4.23 | 0.01, 2,232 | 0.6 | 0.01 | 0.00, 2.99 | 0.13 |
| Other | 0.02 | 0.00, 8.52 | 0.2 | 2.98 | 0.00, 440,468 | 0.9 | 0.53 | 0.00, 719 | 0.9 |
| *NIHSS upon admission* | 1.05 | 0.95, 1.17 | 0.4 | 1.06 | 0.84, 1.36 | 0.6 | 1 | 0.84, 1.19 | >0.9 |
| *ASPECTS value* | — | — | — | — | — | — | — | — | — |
| 10 | — | — | — | — | — | — | — | — | — |
| 6 | 0.8 | 0.04, 11.3 | 0.9 | 0 | — | >0.9 | 0 | — | >0.9 |
| 7 | 0.4 | 0.03, 6.60 | 0.5 | 56.4 | 1.03, 18,964 | 0.083 | 0.03 | 0.00, 0.89 | 0.093 |
| 8 | 0.27 | 0.04, 1.32 | 0.12 | 2.11 | 0.08, 50.3 | 0.6 | 7.01 | 0.84, 81.3 | 0.087 |
| 9 | 0.02 | 0.00, 0.18 | 0.002 | 4.35 | 0.06, 326 | 0.5 | 0.37 | 0.02, 4.44 | 0.5 |
| *Atrial fibrillation* | 0.96 | 0.21, 4.70 | >0.9 | 1.07 | 0.06, 18.1 | >0.9 | 1.76 | 0.27, 11.2 | 0.5 |
| *Hypertension* | 1.46 | 0.31, 6.75 | 0.6 | 0.02 | 0.00, 0.89 | 0.11 | 0.96 | 0.11, 7.38 | >0.9 |
| *Hyperlipidemia* | 2.75 | 0.50, 16.8 | 0.3 | 0.08 | 0.00, 2.12 | 0.2 | 38.6 | 3.44, 903 | 0.008 |
| *Diabetes mellitus* | 1.29 | 0.29, 5.83 | 0.7 | 27.1 | 0.87, 3,678 | 0.1 | 0.47 | 0.03, 5.62 | 0.6 |
| *History of ischemic stroke* | 15.9 | 1.40, 284 | 0.036 | 3.53 | 0.17, 119 | 0.4 | 10.5 | 0.74, 175 | 0.078 |
| *Premorbid mRS* | — | — | — | — | — | — | — | — | — |
| 0 | — | — | — | — | — | — | — | — | — |
| 1 | 0.4 | 0.07, 1.96 | 0.3 | 103 | 3.29, 31,671 | 0.032 | 0.3 | 0.03, 2.60 | 0.3 |
| *Site of intracranial vessel occlusion* | 0.85 | 0.30, 2.44 | 0.8 | 2.18 | 0.22, 36.7 | 0.5 | 1.82 | 0.38, 11.1 | 0.5 |
| *Intravenous thrombolytic use* | 2.93 | 0.72, 13.5 | 0.14 | 15.3 | 0.36, 4,056 | 0.2 | 1.48 | 0.26, 8.71 | 0.7 |
| Multivariable logistic regression models adjusted for listed covariates. | | | | | | | | | |
| ASPECTS = Alberta Stroke Program Early CT Score; CI = confidence interval; FPE = first-pass effect (mTICI ≥2c after single pass); mRS = modified Rankin Scale; NIHSS = National Institutes of Health Stroke Scale; OR = odds ratio. | | | | | | | | | |
| Reference categories: Race (Asian), ASPECTS value (10), premorbid mRS (0). | | | | | | | | | |
